# EEG correlates of Fitts’s law during preparation for action

**DOI:** 10.1007/s00426-012-0418-z

**Published:** 2012-02-05

**Authors:** D. Kourtis, N. Sebanz, G. Knoblich

**Affiliations:** 1 Donders Institute for Brain, Cognition and Behaviour, Radboud University Nijmegen, Nijmegen, The Netherlands; 2 Department of Cognitive Science, Central European University, Budapest, Hungary

## Abstract

Humans’ inability to move fast and accurately at the same time is expressed in Fitts’s law. It states that the movement time between targets depends on the index of difficulty, which is a function of the target width and the inter-target distance. The present study investigated the electrophysiological correlates of Fitts’s law during action planning using high-density electroencephalography. Movement times were scaled according to Fitts’s law, indicating that participants could not overcome the speed–accuracy trade-off during a 1-s preparation period. Importantly, the index of difficulty of the planned movement correlated linearly with the amplitudes of the cognitive N2 and P3b components, which developed during the planning period over parieto-occipital areas. These results suggest that the difficulty of a movement during action planning is represented at a level where perceptual information about the difficulty of the ensuing action is linked to motor programming of the required movement.

## Introduction

It is common experience in everyday life that the accuracy of performing actions, such as inserting a key into a lock or making a basketball shot, is inversely related to the speed of execution. The inability to act fast and accurately at the same time constitutes one of the basic principles of motor control that is often referred to as the speed–accuracy trade-off. This principle formed the basis for the formulation of Fitts’s law (Fitts, 1954), which states that the time needed to move as fast as possible between two targets is a function of the width of the targets and the distance separating them. Fitts’s law is expressed mathematically by the following formula:

$$ {\text{MT }} = \, a \, + \, b{\text{ ID}} $$

where MT denotes movement time, ID the index of difficulty of the movement and *a* and *b* are empirical constants. The critical variable is the ID, which depends on the amplitude (*A*) of the movement (i.e., the distance separating the targets) and the width (*W*) of the targets. It is expressed mathematically as:

$$ {\text{ID }} = \, \log_{2} \left( {2A/W} \right) $$

The above formula states that moving fast and accurately between targets becomes more difficult as the distance between the targets gets larger and/or as the width of the targets gets smaller, and that there is a fixed relation between movement amplitude and target width, which defines a particular index of movement difficulty.

Fitts’s law has been extensively studied since its introduction and it has been proven to be one of the most robust laws in motor control, which holds for different movement types, contexts and movement effectors (e.g., Bakker, de Lange, Stevens, Toni, & Bloem, 2007; Decety & Michel, 1989; Maruff & Velakoulis, 2000; Plamondon & Alimi, 1997; Wu, Yang, & Honda, 2010). There are only few situations where Fitts’s law is modulated, such as the performance of rapid cyclic movements (Smits-Engelsman, Van Galen, & Duysens, 2002) or the placement of the targets in structural perceptual arrays (Pratt, Adam, & Fischer, 2007). The underlying mechanism of such deviation from Fitts’s law seems to be rooted in the motor system in the way it exploits its physiological properties (Smits-Engelsman et al., 2002) and utilizes perceptual information (Radulescu, Al-Aidroos, Adam, Fischer, & Pratt, 2011).

It has also been shown that Fitts’s law holds when people judge whether a movement is feasible for another person (Grosjean, Shiffrar, & Knoblich, 2007), except observers with brain damage in frontal brain areas supporting action planning (Eskenazi, Grosjean, Humphreys, & Knoblich, 2009). However, less is known about how individuals prepare to perform a task if both speed and accuracy are required. Although there is evidence that people have an inherent knowledge of Fitts’s law before action initiation (Augustyn & Rosenbaum, 2005; Bertucco & Cesari, 2010), the processes that allow actors to derive such knowledge prior to the action have not been studied in detail.

### Do action plans specify movement difficulty?

The objective of our study was to elucidate the operation of these processes by means of high-density electroencephalography (EEG). In particular, we aimed to explore the possibility that prospective actors represent in advance the difficulty of actions to be performed. We hypothesized that people’s action plans not only specify obvious parameters of the movement, such as which effector to use and which location in space to target, but also the difficulty of the action, in terms of the ID of Fitts’s law. This would indicate that people’s action planning can be informed by their own motor system and it could be achieved using internal models, which are believed to be utilized by the central nervous system (CNS) to internally simulate an action and the consequences it causes in the environment (Kawato, Furukawa, & Suzuki, 1987; Wolpert, Ghahramani, & Jordan, 1995). Internal models can be broadly classified into forward and inverse internal models (Desmurget & Grafton, 2000; Wolpert & Miall, 1996). Forward models rely on the notion of an “efferent copy” of the motor command, based on which the CNS predicts the sensory consequences of a particular action (Jordan & Rumelhart, 1992; Wolpert & Miall, 1996). Following execution, the actual consequences of the action are compared with the predicted ones and the motor plan can be adjusted accordingly.

Challenging this view, the proponents of inverse internal models assume an a priori motor plan and consider the current motor state (e.g., hand posture) as the input that is used by the model to estimate the motor command which resulted in that particular motor state (Atkeson, 1989; Lacquaniti, Borghese, & Carrozzo, 1992). There are, however, a number of studies that point towards the existence of hybrid internal models, which consist of a forward model that broadly specifies a motor plan, which is continually updated and refined in real time by internal feedback loops (Bhushan & Shadmehr, 1999; Desmurget and Grafton 2000; Hoff & Arbib, 1993).

### The current study

To investigate whether simulation of an action involves predicting the difficulty of a movement, we asked participants to prepare movements specified by a fully informative visual cue, followed 1000 ms later by a go signal that prompted the participants to actually perform the prepared movement. Similar to the original setup used by Fitts (Fitts, 1954), the participants performed uni-manual (left or right) tapping movements holding an electronic stylus on rectangular targets drawn on paper sheets, which were placed on digitizer tablets. We included three different target sizes, whose distances from the starting position were arranged in such a way as to represent three different levels of movement difficulty as defined by Fitts’s law. Our EEG analysis was focused on preparation interval, the time period between cue and go signal onset (foreperiod), during which the participants were motionless but preparing to perform the action indicated by the cue in a given trial.

To our knowledge, so far Fitts’s law has not been investigated with EEG. Accordingly, there are no event-related potentials (ERPs) that have been previously associated with the index of movement difficulty (ID). Nevertheless, the theories described above allow us to make predictions about which parameters may correlate with preparing movements differing in ID. Thus, the focus of our analyses was on ERPs that were typically linked with updating of internal models, decision-making and motor preparation.

First, we examined the amplitude of the parietal P3b component (or “classical P300″), which is an endogenous, cognitive potential, peaking approximately 300 ms after the presentation of a stimulus. The most popular view regarding the functional significance of the P3b is that it reflects the updating of an internal model in working memory in response to task-relevant stimuli (Donchin & Coles, 1988; Polich, 2007). However, more recent accounts (Nieuwenhuis, Aston-Jones, & Cohen, 2005; Verleger, Jaśkowski, & Wascher, 2005) suggest that the P3b is indirectly related to decision-making, facilitating the organization of the appropriate response. It has been proposed that the P3b may act as a “bridge”, linking perception and action by monitoring the decision-making process (Verleger, 2008; Verleger, Jaśkowski, & Wascher, 2005). Both theories, however, postulate that stimulus evaluation and categorization processes precede the processes reflected in the P3b. In the pre-cueing interval of the present experiment, the P3b may either reflect a process of updating memory (Polich, 2007) with particular parameters of an ensuing movement or it reflect monitoring of the decision to prepare a movement with a predefined index of difficulty (Verleger, 2008). Especially the latter account would predict that ID modulates the amplitude of the P3b.

Furthermore, we investigated whether people simulated performing the action using their motor system during the preparation interval. Previous studies have shown that Fitts’s law holds also for action perception (Grosjean, Shiffrar, & Knoblich, 2007) and action imagery (Decety & Jeannerod, 1995), suggesting that people are capable of simulating the index of difficulty of a movement without actually performing the movement. Thus, it is well possible that such a motor simulation takes place when specifying the plan to perform particular actions. In order to determine whether a motor simulation takes place, we examined the amplitude of motor-related ERPs, which typically develop during the delay period between the cue and the go stimulus. We examined the amplitude of the contingent negative variation (CNV) (Walter, Winter, Cooper, McCallum, & Aldridge, 1964) and the lateralized readiness potential (LRP) (cf. Coles, 1989). Both reflect movement preparatory activity generated mostly in the premotor and primary motor areas, respectively (Leuthold, Sommer, & Ulrich, 2004). Our prediction was that if participants engage in motor simulation before movement onset, the amplitude of the CNV and/or the LRP should be modulated according to Fitts’s law. In this case, an interesting question was whether the participants would be able to at least partially overcome the constraints of the speed–accuracy trade-off, deviating from the predictions for movement times as imposed by Fitts’s law.

## Methods

### Participants

Continuous EEG data were recorded form 17 right-handed participants (12 females and 5 males; age = 22.6 ± 3.4 yrs). All participants had normal or corrected-to-normal vision and had no history of hand or arm injuries or diseases, or any mental, cognitive and other neurological disorder. All participants provided their informed consent.

### Experimental setup and procedure

**Fig. 1:**
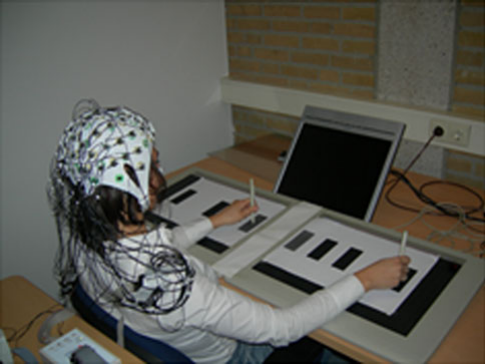
Experimental setup: the participant performs a tapping movement with her right arm towards the far right target while her left arm remains still. The figure displays a movement performed in a block containing the large targets layout (i.e., 4-cm width). The setup of the blocks containing the narrow (i.e., 1-cm width) and the medium sized (i.e., 2-cm width) targets differed from the figure only with regard to the distances between the targets and the “start area” (see Table 1)

The experiment was run in a quiet, normally illuminated room. The participants were seated comfortably in front of a table, where two digitizer tablets (WACOM Ltd, Model UD-1218-RE) were placed adjacent to each other. On each tablet, a sheet of A3 paper was placed with one grey (“start”) and three equally sized black rectangles (“targets”) printed on its surface (Fig. 1). An inclined computer screen was placed centrally in front of the participants at an approximate distance of 100 cm (Fig. 1).

**Fig. 2:**
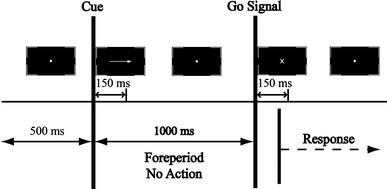
Example of a trial: the trial started with the presentation of a dot for 500 ms, directing the participant’s gaze to the cue location. Then, an *arrow* was displayed for 150 ms, indicating the amplitude of the to-be-performed movement (middle of the target array in the example above). The participants were instructed to withhold their response, while fixating on the *dot*, until the go signal appeared on the screen

The experimental task was a pre-cueing task where visual cues presented on a computer screen specified a movement to be performed after a preparation period. At the beginning of each trial, the participants held using a precisions grip two electronic styli (one in each hand) placed on the middle of the grey rectangular ‘start areas’. The cue stimulus, consisting of a small, medium or large directional arrow, designated a target located on two sheets of papers placed on two digitizer tablets to the left and the right of the participant. Following a foreperiod of 1000 ms, an imperative go signal prompted the participants to perform a swift and accurate, unimanual tapping movement using the electronic stylus on the surface of the designated target and to then return back to the starting position (Fig. 2).

The experiment was divided into 12 blocks that lasted 4 min and 12 s each, preceded by a practice block of equal duration. The order of blocks was randomized across participants. Each block consisted of 63 trials. The width of the target areas was kept constant within each block of trials. Three different widths were used: 1, 2 and 4 cm. The centre-to-centre distances of the targets with the “start” rectangles were arranged in such a way as to correspond to three different movement IDs as defined by Fitts’s law (Table 1).

**Table 1:** Movement amplitudes, target widths and resulting IDs in the present experiment

Movement amplitudes			
Target width (cm)	ID		
	2 (cm)	3 (cm)	4 (cm)
1	2	4	8
2	4	8	16
4	8	16	32

All visual stimuli were of white colour presented over a black background and were enclosed in white square brackets of 4^o^ width and 2.58^o^ height of visual angle. Each trial started with the display of a fixation dot with a diameter of 0.02^o^ visual angle for 500 ms. It was followed by the difficulty cue that consisted of three different sized white directional arrows (0.86^o^, 1.72^o^ and 3.44^o^ of visual angle) enclosed by white square brackets of 4.00^o^ width and 2.58^o^ height of visual angle presented over a black background. The arrows pointed to each of the six targets (three on the left, three on the right) with equal probability (14.3% of the trials). In addition, a no-go stimulus, consisting of the letter “o” (diameter 0.40^o^ of visual angle) was displayed on the remaining 14.3% of the trials. The difficulty cue was displayed for 150 ms. After a period of 850 ms during which the fixation dot was displayed, an imperative go signal consisting of the letter “x” (0.40^o^ of visual angle) was displayed for 150 ms. The inter-stimulus interval and the inter-trial interval were kept constant across the experiment at 1000 ms and 4000 ms, respectively.

### Data acquisition

Although Fitts’s law describes the effect of speed–accuracy trade-off on movement times, we also examined the reaction times of our participants, exploring the possibility that a prospective “difficult” movement might slow down movement initiation. Reaction time was defined as the time interval between the onset of the go signal and the release of the electronic stylus from the surface of the “start” rectangle. Movement time was defined as the time interval between the release of the electronic stylus from the surface of the “start” rectangle and the subsequent tapping on the surface of the designated “target” rectangle. For each participant, all reaction and movement times that were smaller than 100 ms or differed by more than 2 standard deviations (SD) from the means within each condition were removed from further analysis.

EEG was recorded continuously with Ag/AgCl electrodes from 64 scalp electrodes relative to an (off-line) average mastoid reference. The electrodes were placed according to the International 10–20 Electrode System (American Electroencephalographic Society, 1994 **)** using a carefully positioned nylon cap. Vertical eye movements were monitored using a pair of bipolar electro-oculography (EOG) electrodes positioned directly above and under the right eye, while horizontal eye movements were monitored using a pair of bipolar electro-oculography (EOG) electrodes positioned at the outside of each of the eyes.

### Data processing and analysis

EEG data processing was performed off-line using the Brain Vision Analyzer (V. 1.05, Brain Products GmbH, Gilching, Germany) software. Initially, ocular correction using the Gratton–Coles algorithm (Gratton, Coles, & Donchin, 1983) implemented in Brain Vision Analyzer was used to eliminate or reduce artefacts induced by horizontal or vertical eye movements. The corrected EEG data were then segmented off-line in epochs from 300 ms before cue onset to 1500 ms after cue onset. The data were filtered using a high-pass filter of 0.05 Hz (24 dB/octave) and a low-pass filter of 60 Hz (24 dB/octave) to remove slow drifts and excessive noise, respectively. Individual trials were removed before averaging if they contained artefacts induced by vertical or horizontal eye movements, which were not entirely removed during ocular correction, or further artefacts possibly induced by body, head or arm movements. The rejection criterion was that the difference between the maximum and the minimum value within a given segment exceeded 100 μV. Data from individual trials containing early or incorrect responses were also removed before averaging. Averages were separately constructed for each subject and each condition. The last 200 ms before cue onset was considered as baseline period. Event-related potential amplitudes were analysed by pooling the values of neighbouring electrodes within regions of interest, identified on the basis of scalp topographies (see “Results”).

## Results

### Behavioural analysis

#### Reaction times

For left hand responses, the reaction times (RTs) were 363 ± 67 ms, 370 ± 77 ms and 363 ± 77 ms for movement ID2, ID3 and ID4, respectively. For right hand responses, the RTs were 346 ± 63 ms, 363 ± 72 ms and 353 ± 77 ms for movement ID2, ID3 and ID4, respectively. The RTs were generally shorter for right hand responses (t(16) = 3.204, p = 0.006), which was expected because we tested only right-handed participants. However, there was no consistent increase of RTs as a function of ID.

#### Movement times

**Fig. 3:**
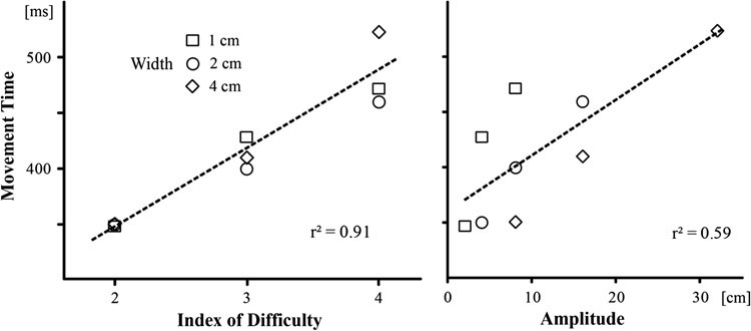
Mean movement time as a function of movement ID (*left*) and movement amplitude (*right*)

The analysis of movement times (MTs) showed no difference between left (417 ± 48 ms) and right hand (414 ± 49 ms) responses (t(16) = 0.946, p = 0.358). Therefore, the ensuing analysis was performed on pooled data from both hands. Consistent with the predictions of Fitts’s law, the MTs increased linearly with increasing movement ID (Fig. 3, left). The regression analysis yielded a significant *r* ^2^ = 0.91 (*F*(1,16) = 70.6, *p* < 0.001) and the following regression equation: MT (ms) = 211 + 68 ID.

Since larger IDs are by default related to larger movement amplitudes, we examined whether the increase in movement times could be equally well or even better predicted by movement amplitude. The regression analysis showed that amplitude is a weaker predictor: *r* ^2^ = 0.59 (*F*(1,16) = 9.9, *p* = 0.016), because it does not take into account the influence of target width (Fig. 3, right). The resulting following regression equation was MT (ms) = 360 + 5 amplitude.

#### Error rates

The analysis of the error rates showed that the task was performed with remarkable accuracy. In the blocks with the large targets layout, the participants failed to tap within the designated target areas in 0.3, 0.3 and 0.1% of the trials for far, middle and near-located targets, respectively. In the blocks with the medium targets layout, the participants failed to tap within the designated target areas in 0.8, 0.5 and 0.3% of the trials for far, middle and near-located targets, respectively. In the blocks with the small targets layout, the participants failed to tap within the designated target areas in 1.8, 2.0 and 0.7% of the trials for far, middle and near-located targets, respectively. Although there was a slight increase in error rates with increasing movement ID, the correlation between these two parameters was quite weak (*r* ^2^ = 0.14). Thus, error rated did not clearly follow Fitts’s law. In addition in very few occasions, the participants performed a tapping movement at the wrong target, possibly due to misidentification of the cue stimulus. This error occurred in 0.2, 0.2 and 0.1% of the trials following a large, medium and small arrow, respectively.

#### EEG analysis

**Fig. 4:**
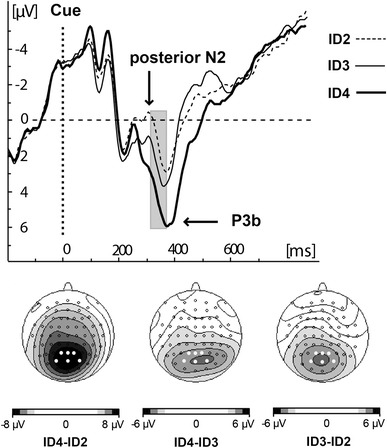
Grand average waveforms derived from pooled electrode sites Pz, P1, P2, POz, PO3 and PO4, highlighted as *black circles* and scalp voltage distributions of the difference between different indices of movement Difficulty in the time intervals from 310 to 370 ms after cue onset. The *grey bar* indicates the latency window for amplitude analysis

The EEG analysis showed that increasing movement IDs were accompanied by decreasing amplitudes of a mid-parieto-occipital component of negative polarity peaking around 310 ms after cue onset (it will henceforth be denoted as “posterior N2”) and increasing amplitudes of the centro-parietal P3b component peaking around 370 ms after cue onset. A detailed examination of the data showed that the linear relation between movement IDs and ERPs amplitude was maximum around 340 ms after cue onset, halfway between the posterior N2 and P3b peaks. Thus, the posterior N2/P3b amplitude was evaluated by pooling the mean activity between 310 and 370 ms after cue onset from electrodes Pz, P1, P2, POz, PO3 and PO4, where the difference between IDs was most pronounced (Fig. 4).

**Fig. 5:**
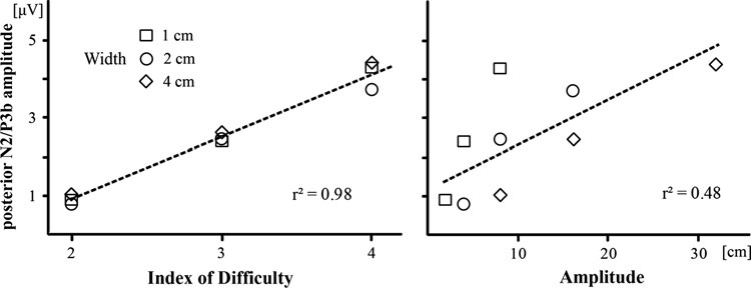
Mean posterior N2/P3b amplitude between 310 and 350 ms after cue onset (electrodes Pz, P1, P2, POz, PO3 and PO4) as a function of movement ID (*left*) and movement amplitude (*right*)

The regression analysis yielded a significant *r* ^2^ = 0.98 (*F*(1,16) = 312.7, *p* < 0.001) and the following regression equation: posterior N2/P3b (μV) = −2.3 + 1.6 ID (Fig. 5, left). We also examined the relation of movement amplitude to the amplitude of the posterior N2/P3b components. Similarly to the movement times analysis, the movement amplitude was a weaker predictor *r* ^2^ = 0.48 (*F*(1,16) = 6.4, *p* = 0.040) compared to movement IDs, because the target width was not taken into account (Fig. 5, right).

The resulting following regression equation was posterior N2/P3b (μV) = 1.4 + 0.1 amplitude.

**Fig. 6:**
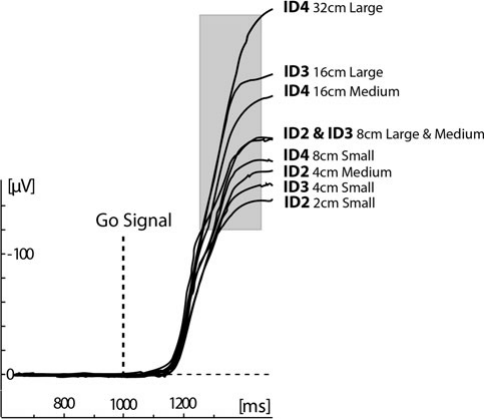
Grand average waveforms depicting horizontal eye movements in all conditions derived from the bipolar set of electrodes placed at the outside of each eye. The distances in centimetre refer to the movement amplitude and the words “large”, “medium” and “small” to the target size. The *grey bar* indicates the latency window for amplitude analysis

A potential problem in our design was that the participants were instructed to look at the cue-designated target in order to be accurate. However, eye movements occurred after the go signal. Furthermore, previous studies have shown that saccadic eye movements do not follow Fitts’s law (Chi & Lin, 1997). Nevertheless, we performed an additional analysis to exclude any possible relation between the posterior N2/P3b amplitude and the subsequent eye movements. In this analysis, we focused on horizontal eye movements, because the vertical distance between each target and cue location was constant; therefore, vertical eye movements were of the same amplitude, irrespective of target width and location. Since it is known that eye gaze arrives at a target approximately at response onset (Prablanc, Echallier, Komilis, & Jeannerod, 1979; Prablanc & Martin, 1992), we quantified eye movement amplitude as the mean lateralized activity recorded from the bipolar electrodes located at the side of each eye (Lateralized HEOG) between 250 and 450 ms (Fig. 6) after go signal onset (i.e., mean RTs ≈350 ms).

**Fig. 7:**
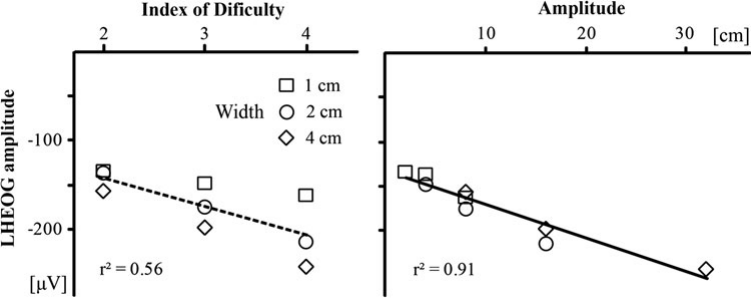
Mean lateralized HEOG amplitude between 250 and 450 ms after go signal onset as a function of movement ID (*left*) and movement amplitude (*right*)

The results showed a different pattern from the analyses of movement times. More specifically, the stronger predictor for lateralized horizontal eye movement amplitude was the movement amplitude (Fig. 7, left). The regression analysis yielded a significant *r* ^2^ = 0.91 (*F*(1,16) = 74.7, *p* < 0.001) and the following regression equation: LHEOG (μV) = −133 − 3.8 amplitude. In comparison, the regression analysis for movement ID yielded much weaker results, *r* ^2^ = 0.56 (*F*(1,16) = 8.8, *p* = 0.021) compared to movement amplitude, and the resulting regression equation was LHEOG (μV) = −77 − 32 ID (Fig. 7, right). This clearly shows that (horizontal) eye movements did not follow Fitts’s law, so we can safely exclude the possibility that the posterior N2 and P3b have any connection with preparatory eye movement.

**Fig. 8:**
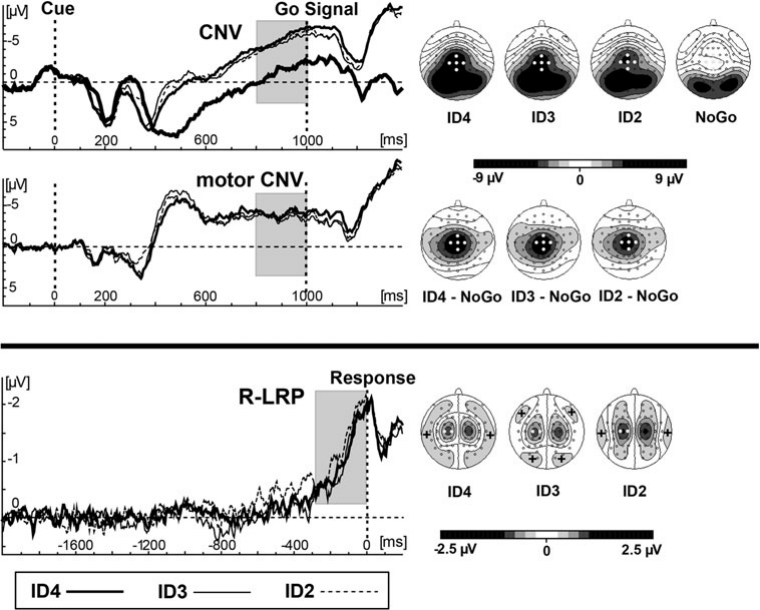
*Top* grand average waveforms derived from pooled electrode sites Cz, C1, C2, FCz and CPz, highlighted as *white circles* and scalp voltage distributions during the last 200 ms before the go signal onset. The “motor CNV” was computed by subtracting the CNV in the NoGo condition from the CNV in each of the action planning conditions. *Bottom* grand average waveforms derived from pooled electrode pairs C1/2 and C3/C4, highlighted as *white circles* and scalp voltage distributions of lateralized activity during the last 200 ms before response onset. The *grey bars* indicate the latency windows for amplitude analysis. The *grey-shaded areas* at all scalp topographies denote scalp activity of negative polarity, unless the “+” sign is included, which denotes activity of positive polarity

Finally, we examined the amplitude of two potentials that are widely observed when people prepare to perform a movement, the motor CNV and the response locked LRP (R-LRP). The former component is believed to reflect primarily activity from the supplementary motor area, whereas the latter is believed to reflect activity from the primary motor cortex. Prior to the analysis of the CNV, to remove activity due to stimulus anticipation processes and to only keep the motor part of the CNV, we subtracted the CNV in the no-go condition from the CNV in the action planning conditions. The motor CNV was quantified as the mean activity from electrodes Cz, FCz, CPz, C1 and C2 during the last 200 ms before the go signal onset. The LRP was quantified as the mean activity from electrode pairs C1/2 and C3/4 during the last 200 ms before response onset (Fig. 8). Two regression analyses showed that neither the motor CNV nor the LRP showed any modulation according to the movement ID or the movement amplitude (*p*s > 0.17).

## Discussion

The objective of our study was to examine the effect of action planning on the speed–accuracy trade-off as expressed by Fitts’s law and to investigate the brain processes that allow individuals to represent in advance difficulty of a prospective action. Our results showed that despite the 1-s preparation interval, participants were not able to overcome the restrictions imposed by the speed–accuracy trade-off. Their movement times were scaled according to the predictions of Fitts’s law. Moreover, our EEG analysis showed that the ID of a planned movement was represented at an intermediate stage between perception and action, because the amplitude of the posterior N2 and P3b components, elicited by the cue that specified movement difficulty, were modulated according to Fitts’s law. In contrast, ERP components, which indicate that a motor simulation takes place during the preparation interval (i.e., motor CNV, LRP), were not significantly affected by the movement ID.

Previous studies have found that people have an inherent knowledge of Fitts’s law during action planning. This has manifested itself behaviourally either as selection of the optimal starting position (Augustyn & Rosenbaum, 2005) or as anticipatory postural adjustments before movement onset (Bertucco & Cesari, 2010). Further studies addressing the importance of allocentric information in rapid aiming movements argued that the locus of Fitts’s law effects lies in the planning phase of the movement (Bradi, Adam, Fischer, & Pratt, 2009) and probably after perceptual processing (Radulescu et al., 2011). In line with these claims, the amplitude of the cue-induced posterior N2 and P3b components in the present study were strongly correlated to the ID of the prospective movement. This finding suggests that the participants had processed the information conveyed by the difficulty cue and represented the movement ID well before movement onset.

The modulation of the P3b was predicted on the basis of its proposed functional role of updating internal models of the environment (Donchin & Coles, 1988; Krigolson, Holroyd, van Gyn, & Heath, 2008), possibly also reflecting decision-making processes (Nieuwenhuis, Aston-Jones, & Cohen, 2005; Verleger, Jaśkowski, & Wascher, 2005). In terms of the neural networks underlying these functions, it is widely believed that the P3b is generated in the parietal lobe (Bledowski, Prvulovic, Hoechstetter, Scherg, Wibral, Goebel, & Linden, 2004; Ford, Sullivan, Marsh, White, Lim, & Pfefferbaum, 1994; Polich, 2007; Verleger, 2008), which is often considered to be an integral part in forming “intentions or high-level cognitive plans for movement” (Andersen & Buneo, 2002). The findings of a large number of studies strongly suggest that the parietal lobe (along with the cerebellum) has a crucial role in forming and updating forward (i.e., predictive) internal models of action (Blakemore & Sirigu, 2003; Desmurget & Grafton, 2000). Moreover, the parietal lobe is considered to act as a “neural” comparator computing the error between the predicted and the actual motor state, facilitating the updating of muscle activation patterns (Desmurget, Epstein, Turner, Prablanc, Alexander, & Grafton, 1999). Interestingly, the P3b has also been associated with coding the discrepancy (i.e., error) between the actual and predicted motor command (Krigolson et al., 2008), and also with the probability of the response outcome (Hajcak, Holroyd, Moser, & Simons, 2005; Hajcak, Moser, Holroyd, & Simons, 2007). It is thus possible that, in the present study, the higher P3b before movement with higher IDs reflects the higher likelihood of committing an error when performing a movement with a higher ID. In other words, the P3b amplitude may reflect the probability of a discrepancy between the planned and the actual movement.

Alternatively, it may be argued that the P3b modulation reflected the difficulty of correctly identifying the (arrow) cue signal. In other words, it may be the case that the length of the arrow made the cue more or less salient, affecting thus the P3b amplitude (cf. Johnson, 1986). However, the arrow cues were presented in the complete absence of any distractors and always at the same location of the screen. Moreover, the participants did not report any problems in identifying them during the practice and experimental blocks and committed a remarkably low number of errors. It should be pointed out that the participants tapped very scarcely at the wrong target (~0.2% of the trials). This occurred equally often for different cues (the long, medium or small arrow). Thus, it seems unlikely that the present P3b modulation reflects a differential difficulty of processing stimulus features.

In addition to the predicted effect on the P3b, we observed an amplitude modulation of the preceding posterior N2 component, which peaked around 310 ms over mid-parieto-occipital sites and was inversely related to the ID of a prospective movement. Posterior negative ERPs of similar latency are typically observed in visual classification and search tasks. Probably the most studied one is the N2pc, which is an enhanced posterior negativity contralateral to the visual field of attended stimuli (Luck & Hillyard, 1994a, b) and it is believed to reflect spatial filtering processes and possibly the rapid attentional selection of visual target objects (Eimer & Kiss, 2010). However, we observed no lateralized activity around the latency of the posterior N2; hence, any association between the posterior N2 and the N2pc is highly unlikely.

In addition to the N2pc, a bilateral posterior component occurring in the same time range, often termed N2pb (Luck & Hillyard, 1994a) or N2p (Schubö, Meinecke, & Schröger, 2001), is considered to reflect stimulus selection, analysis and classification processes (Akyürek, Dinkelbach, Schubö, & Müller, 2011; Schubö, 2009; Schubö et al., 2001; Schubö, Schröger, & Meinecke, 2004). The N2p amplitude decreases with increasing target stimulus eccentricity (Schaffer, Schubö, & Meinecke, 2011) and, given that in the present study there was a direct relation between arrow length and movement ID, it is possible that the modulation of the posterior N2 simply reflects the distance of the arrowhead of each cue stimulus from the centre of the display screen. However, it has to be noted that the N2p is typically larger over lateral areas (i.e., PO7/PO8 electrode in Schaffer et al., 2011), whereas the posterior N2 in the present study was larger over or very close to the midline.

An alternative account regarding the functional role of the posterior N2 can be derived from studies, which investigated the electrophysiological correlates of reaching preparation. Praamstra, Kourtis, and Nazarpour (2009) studied reaching preparation towards multiple potential targets and argued that the midline posterior N2 reflected the evaluation of the stimulus against a visuospatial representation in memory and that it is sensitive to the spatial relation of possible movement directions and targets. In an earlier study where participants performed precise reaching movements (Naranjo, Brovelli, Longo, Budai, Kristeva, & Battaglini, 2007), the (midline) posterior N2 was localized to the superior parietal lobule/precuneus, which has been suggested as the human homologue of the parietal reach region that has been extensively studied in monkeys (Astafiev, Shulman, Stanley, Snyder, Van Essen, & Corbetta, 2003; Connolly, Andersen, & Goodale, 2003). The authors argued that posterior N2 reflected movement selection processes: in other words, “the computation of the motor plan”. Accordingly, the posterior N2 in our study may reflect the evaluation/classification of the cue stimulus based on the movement difficulty of the associated action. Although this is a preferred interpretation, we cannot fully rule out an alternative interpretation based on stimulus classification.

In contrast to the scaling of parietal activity, we found no evidence of an analogous scaling of motor cortex activation prior to movement in the amplitude of the motor CNV and the LRP. The absence of such a scaling is rather surprising when considering the rather large number of mental imagery studies, which have consistently showed that imagined movements follow the constraints described by Fitts’s law in a similar way to real movements (e.g., Decety & Jeannerod, 1995; Maruff & Velakoulis, 2000; Slifkin & Grilli, 2006). In addition, it was found that people judge the perceived difficulty of an observed action according to Fitts’s law (Grosjean et al., 2007) using their motor system (Eskenazi, Rothstein, Grosjean, & Knoblich, 2012). Taking into account the anticipatory nature of action simulation (e.g., Kilner, Vargas, Duval, Blakemore, & Sirigu, 2004), one would expect that the difficulty of the subject to be prepared for the movement would affect the amplitude of the motor CNV and/or the LRP. Instead, our analyses showed that the pre-movement amplitude of these components was unaffected by movement ID and movement amplitude, indicating a constant, generic state of motor preparation irrespective of the details of a given movement.

The present results are in line with the notion of hybrid internal models of action, which postulate the formation of a “crude” motor plan before action onset, which gets constantly updated through feedback loops during movement execution (Desmurget & Grafton, 2000). The importance of such feedback loops in Fitts’s law tasks is supported by findings that provided support for dynamic theories of perception and action (Schöner & Kelso, 1988). Such theories suggest that the scaling of the movement times according to the movement IDs is an emergent process (Mottet, Guiard, Ferrand, & Bootsma, 2001), which occurs during action performance rather than action preparation. Thus, it not only depends on the limited information capacity of the motor system (Fitts & Peterson, 1964), but also on the biomechanical properties of the movement effectors (Smits-Engelsman et al., 2002; Dounskaia, Wisleder, & Johnson, 2005).

Although dynamic accounts of perception and action and the theories of action simulation seem to contradict each other at a first glance, it is likely that they highlight the flexibility of the motor system in planning and executing an action. Such flexibility is supported by the inconsistent results that have been produced in the study of the relation between pre-movement potentials and kinetic and kinematic movement parameters (e.g., Kirsch & Hennighausen, 2010; Kirsch, Hennighausen, & Rösler, 2010; Ray, Slobounov, Mordkoff, Johnston, & Simon, 2000; Sommer, Leuthold, & Ulrich, 1994). More specifically in Fitts’s law tasks, it is plausible that when people are deprived of external feedback, as when explicitly asked to imagine the performance of a task, they are well able to simulate the necessary movement in great detail using their motor system. However, in tasks like ours where people expect that sensory and proprioceptive feedback is available, people seem to prefer to broadly plan an action before its onset and then modify it continuously during action execution.

To conclude, the present study showed that individuals performing a fast tapping task conform to the limitations imposed by the speed–accuracy trade-off as described by Fitts’s law, despite a relatively long preparation period. Our EEG analyses suggest that the index of difficulty of the prospective movement is reflected in the amplitude of the posterior N2 and P3b components, which were elicited by the difficulty cue and may reflect updating internal models or the likelihood of committing a movement error. However, there was no evidence that the motor system simulated in advance the details of the prospective movement. Instead, it seems that the modulation of movement times emerges during movement execution. To our knowledge, the present study is the first one to report a specific correlation between the movement IDs and the amplitudes of event-related potentials. This finding could be beneficial in elucidating the precise cognitive and neural mechanisms of movement, performed under the constraints of the speed–accuracy trade-off.
